# In silico prediction of potential chemical reactions mediated by human enzymes

**DOI:** 10.1186/s12859-018-2194-2

**Published:** 2018-06-13

**Authors:** Myeong-Sang Yu, Hyang-Mi Lee, Aaron Park, Chungoo Park, Hyithaek Ceong, Ki-Hyeong Rhee, Dokyun Na

**Affiliations:** 1 School of Integrative Engineering, Chung-Ang University, Seoul, Republic of Korea; 2 School of Biological Sciences, Chonnam National University, Gwangju, Republic of Korea; 3 Department of Multimedia, Chonnam National University, Yeosu, Republic of Korea; 4 College of Industrial Sciences, Kongju National University, Yesan, Republic of Korea

**Keywords:** In silico model, Enzyme reaction prediction, Drug discovery, Machine learning

## Abstract

**Background:**

Administered drugs are often converted into an ineffective or activated form by enzymes in our body. Conventional in silico prediction approaches focused on therapeutically important enzymes such as CYP450. However, there are more than thousands of different cellular enzymes that potentially convert administered drug into other forms.

**Result:**

We developed an in silico model to predict which of human enzymes including metabolic enzymes as well as CYP450 family can catalyze a given chemical compound. The prediction is based on the chemical and physical similarity between known enzyme substrates and a query chemical compound. Our in silico model was developed using multiple linear regression and the model showed high performance (AUC = 0.896) despite of the large number of enzymes. When evaluated on a test dataset, it also showed significantly high performance (AUC = 0.746). Interestingly, evaluation with literature data showed that our model can be used to predict not only enzymatic reactions but also drug conversion and enzyme inhibition.

**Conclusion:**

Our model was able to predict enzymatic reactions of a query molecule with a high accuracy. This may foster to discover new metabolic routes and to accelerate the computational development of drug candidates by enabling the prediction of the potential conversion of administered drugs into active or inactive forms.

## Background

Enzymes are biological macromolecules that mediate chemical reactions by lowering activation energy barrier. Most of cellular processes including metabolism are mediated by enzymes, and molecules from external environment (usually called as xenobiotics) are modified by enzymatic reactions. In drug discovery, metabolic conversion by cellular enzymes has been studied for decades, because bioavailability, toxicity and pharmacological efficacy are easily affected by enzymatic reactions. There have been many attempts to screen a large number of drug candidates to assess potential modification into an inactive compound by enzymes. For accelerated screening of such enzymatic modifications, computational methods have been developed to predict enzymatic reactions with the advance of computing hardware and efficiency of various algorithms. Computational methods still have limitations such as relatively low prediction accuracy, but in silico approaches are advantageous over experimental approaches such as wide coverage, relatively low cost, and fast prediction [1].

Cytochrome P450 (CYP450) family has been highlighted in drug discovery, because the enzymes in this family are involved in about 75% of drug metabolism [2]. For example, the well-known xenobiotics such as caffeine [3], nicotine [4] and alcohol [5] are substrates of CYP450 enzymes and metabolized in human liver. Recently, various in silico approach techniques have been applied to predict the substrates of CYP450 enzymes [6, 7] and CYP450-mediated metabolism [8]. However, there are other enzymes in human body (25% of the drug metabolism) that can modify xenobiotic compounds in various organs, such as intestine. It is, therefore, necessary to accurately predict the enzymatic reactions that mediate the in vivo conversion of drug compounds. For example, tamoxifen, that is a well-known as anti-cancer agent for breast cancer, is bio-activated by CYP2D6, 2C9 and 3A4 enzymes [9], but is inactivated by flavin-containing monooxygenase (FMO) [10]. Therefore, there is a demand for developing in silico methods to predict enzyme reactions covering most cellular enzymes to accurately assess drug metabolism [11, 12].

In this study, we present an in silico model to predict which of human enzymes are able to catalyze query molecules including not only CYP450 enzymes but also other cellular enzymes. Our in silico model can be useful in screening drug candidates and studying undiscovered biochemical reactions.

## Methods

### Data preparation

**Fig. 1:**
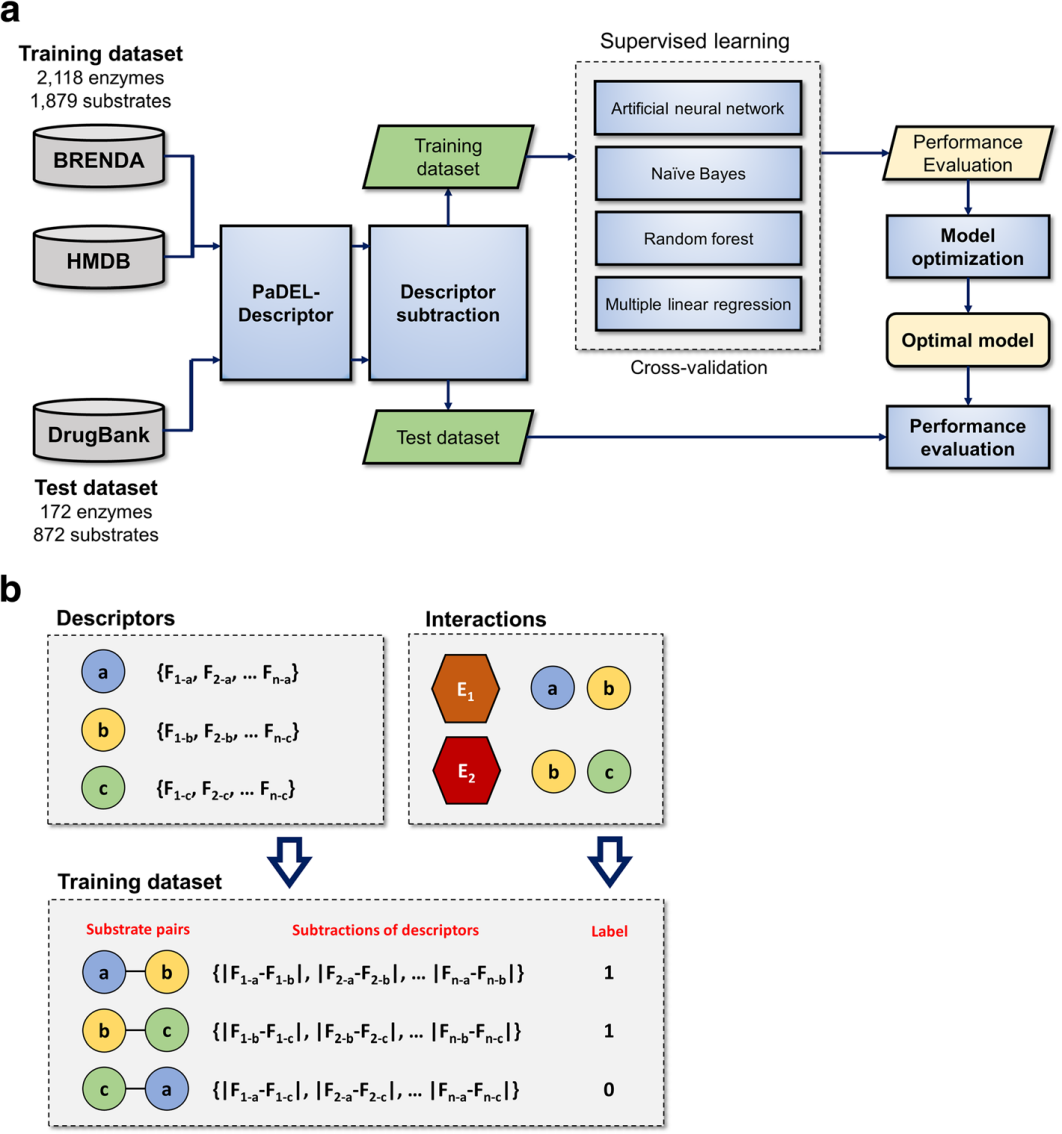
Graphical description for *Methods* **a**. Entire pipeline to construct our prediction model. **b**. A brief graphical description about dataset preparation. 1444 molecular descriptors were calculated for substrates in training dataset, and the subtractions of descriptors were calculated for every substrates pair. For supervised learning, a set of descriptor subtractions of substrates pair was labeled with 1 or 0. *a*, *b* and *c* denote substrates in the training dataset. E1 and E2 denotes enzymes in the dataset

Overall method pipeline is illustrated in Fig. 1a. Human enzymes and their known substrates were extracted from two databases: Human Metabolome Database (HMDB) [13] and BRaunschweig ENzyme DAtabase (BRENDA) [14]. HMDB is a database that contains chemical, clinical and biological information on human metabolites. BRENDA is a curated and a large enzyme database containing various information on enzymatic reactions.

From HMDB 424 substrates and 1449 human enzymes were extracted. From BRENDA 1667 substrates and 1326 enzymes were collected. The two databases were merged and redundant reactions were removed. Accordingly, we obtained 4187 enzyme reactions between 2118 enzymes and 1879 substrates.

### Descriptor calculation

We used PaDEL-Descriptor to calculate chemical and physical properties of substrates [15]. As PaDEL accepts an input molecule expressed in the format of Simplified Molecular-Input Line-Entry System (SMILES), substrate names were converted to SMILES [16]. As HMDB provides substrate names as well as their SMILES, the SMILES were used for PaDEL without modification. For the substrates extracted from BRENDA, their names were firstly converted to into the IUPAC International Chemical Identifier (InChI) [17] and then converted again into SMILES by using ChemSpider [18]. In this study, we used 1444 1-D and 2-D descriptors of the substrates.

### Dataset preparation for machine learning

In this study, we assumed if the physico-chemical properties of a query molecule are similar with those of a substrate, they could be catalyzed by the same enzyme. We calculated the subtractions of 1444 descriptors of every pair of substrates and thereby generated 1879×1878/2 subtracted descriptor values (features). For supervised learning, a set of features calculated between two substrates was labeled with 1 or 0. 1 denotes that the two molecules are catalyzed by the same enzyme, otherwise 0. In our dataset, 11,492 pairs were labeled with 1, and the other 1,752,889 pairs were 0 (Fig. 1b). Each feature was normalized before use.

### Dimensionality reduction

To reduce the number of features in the dataset, we calculated the correlations between a feature and a label (point-biserial coefficient) [19] and then obtained 1444 correlation coefficients. The features were ordered by their absolute value of coefficients and top *n* features were used for training and cross-validation. The number of top features (*n*) was optimized by exhaustive evaluation of the training dataset.

For the correlation calculation, the dataset was divided into two groups by the label. *M*_1_ and *M*_0_ are the averages of a given feature that was labeled as 1 and 0, respectively. *n*_1_ and *n*_0_ are the numbers of the values labeled as 1 and 0, respectively. *n* is the total number of values involved in the feature. *s*_*n*_ denotes a standard deviation, *X*_*i*_ denotes each value, and $\overline{X}$ denotes the average of all the values in the feature. A point-biserial coefficient *r*_*pb*_ was calculated as below.

1 $$ {\displaystyle \begin{array}{c}{r}_{pb}=\frac{M_1-{M}_0}{s_n}\sqrt{\frac{n_1{n}_0}{n^2}}\\ {}\mathrm{where}\ \mathrm{standard}\ \mathrm{deviation}\ {s}_n=\sqrt{\frac{1}{n}\sum \limits_{i=1}^n{\left({X}_i-\overline{X}\right)}^2}\end{array}} $$

### Supervised machine learning

To find the best model, we evaluated four machine learning algorithms (neural network, multiple linear regression, naïve Bayes, and random forest). We used the open source library Orange for the machine learning [20].

### Score-integration

The models firstly predicted whether the two given molecules are catalyzed by the same enzyme based on their subtracted descriptor values. Thus, a query molecule may obtain one or more prediction scores depending on the number of substrates, since an enzyme may have more than one substrates. Therefore, it was necessary to integrate the obtained individual scores. The approaches are an average of all the scores, a maximum score among them, and probability-based scoring method [21]. These scoring methods have their own drawbacks. For example, a simple average may result in a dramatically low score when there are many dissimilar substrates for an enzyme. Thus, we developed an integrated scoring method and compared its performance with other score-integrating methods.

2 $$ {\displaystyle \begin{array}{c}p=\overline{s}+\sum \left({s}_i-\overline{s}\right)\times \sqrt{\frac{\sum_{i=1}^k\ f\left({s}_i-\overline{s}\right)}{k}}\\ {}\mathrm{where}\left\{\begin{array}{c}{\left({s}_i-\overline{s}\right)}^2\\ {}0\end{array}\;\begin{array}{c} if\;{s}_i\ge \overline{s}\\ {} otherwise\end{array}\right.\end{array}} $$

*p* denotes an integrated score, *s*_*i*_ denotes an individual score between a query molecule and a substrate, *k* denotes the number of individual scores larger than the average (${s}_i\ge \overline{s}\Big)$.

Briefly, our integrated scoring method captures the distribution of individual scores by giving a positive weight to the scores higher than their average. For example, a molecule A obtains two scores {0_(A-S1)_, 1_(A-S2)_} and B obtains two scores {0.5_(B-S1)_, 0.5_(B-S2)_} with given two substrates (S1 and S2) catalyzed by enzyme C. Simple average will result in the same integrated score, 0.5. However, it is rational to predict that the molecule A rather than B is catalyzed by the enzyme C due to the high score 1. Our integrated scoring method gives a score of 0.75 and 0.5, respectively, and which indicates that the molecule A is catalyzed by the enzyme C with a higher probability than B. In another example, molecules A and B obtained scores of {0_(A-S1)_, 1_(A-S2)_, 1_(A-S3)_, 1_(A-S4)_} and {0_(B-S1)_, 0.2_(B-S2)_, 0.5_(B-S3)_, 1_(B-S4)_}, respectively, with the four substrates (S1 - S4) catalyzed by enzyme C. Simple maximum may conclude that the two molecules could interact with enzyme C with the same possibility. Intuitively, the molecule A has a higher possibility to react with enzyme C than B. In agreement with the intuition, our method gives a score, 0.86 and 0.61, respectively.

### Performance validation

We divided the dataset into subsets by enzymes, because substrates mediated by the same enzyme would possess very similar physico-chemical properties and therefore substrate-based dataset separation into training and test sets may result in over-fitting. We divided the dataset into 20 subsets by enzymes for 20-fold cross-validation. For further evaluation of the constructed model, we constructed a test dataset from DrugBank, which was not used for the training [22]. DrugBank contains biochemical information of drugs, substrates and their target proteins and we used 872 substrates and 172 enzymes to test our model.

To compare our model with other available prediction methods, we also used the same test dataset: admetSAR [23] and deepDTI [24]. The admetSAR predicts ADMET features of a query molecule. For performance comparison, we queried 872 substrates in our test dataset and obtained their substrate probabilities for CYP2C9, CYP2D6 and CYP3A4. The deepDTI is a deep-belief network-based drug-target interaction prediction tool. As the publicly available software of deepDTI requires training with our own dataset, we firstly trained deepDTI with the training dataset and then the trained model was evaluated on the test dataset.

## Results

### Data construction

To construct a dataset, we compiled human enzymes and their substrates from HMDB and BRENDA databases: 1879 substrates, 2118 enzymes, and 4187 substrate-enzyme reactions. 1,444 molecular descriptors for each substrate, reflecting physicochemical properties, were calculated. For two given chemical compounds, their differences of the 1444 descriptors were calculated to generate features. Consequently, descriptor difference values for 1,764,381 pairs of the substrates were generated and these values were used as features.

We optimized feature number using top 1000 features of the 1444 features to construct the best-performing model. The remaining 444 features were excluded in the model construction due to their zero or very low correlation coefficients < 0.01. In Table 1, the top 10 representative descriptors with high absolute coefficient values are listed, and these descriptors played an important role in the prediction of substrate similarity.

**Table 1:** Top 10 features with a high correlation

Name	R_pb_	Category
minsssCH	−0.0674	Atom type electrotopological state
Hmax	−0.0645	Atom type electrotopological state
SHsOH	−0.0641	Atom type electrotopological state
EE_Dt	−0.0635	Detour matrix
maxHCsatu	−0.0630	Atom type electrotopological state
XLogP	−0.0624	XLogP
CrippenLogP	−0.0619	Crippen logP and MR
Lipoaffinity Index	−0.0618	Atom type electrotopological state
ETA_EtaP_F	−0.0615	Extended topochemical atom
nsOH	−0.0615	Atom type electrotopological state

### Model construction

**Fig. 2:**
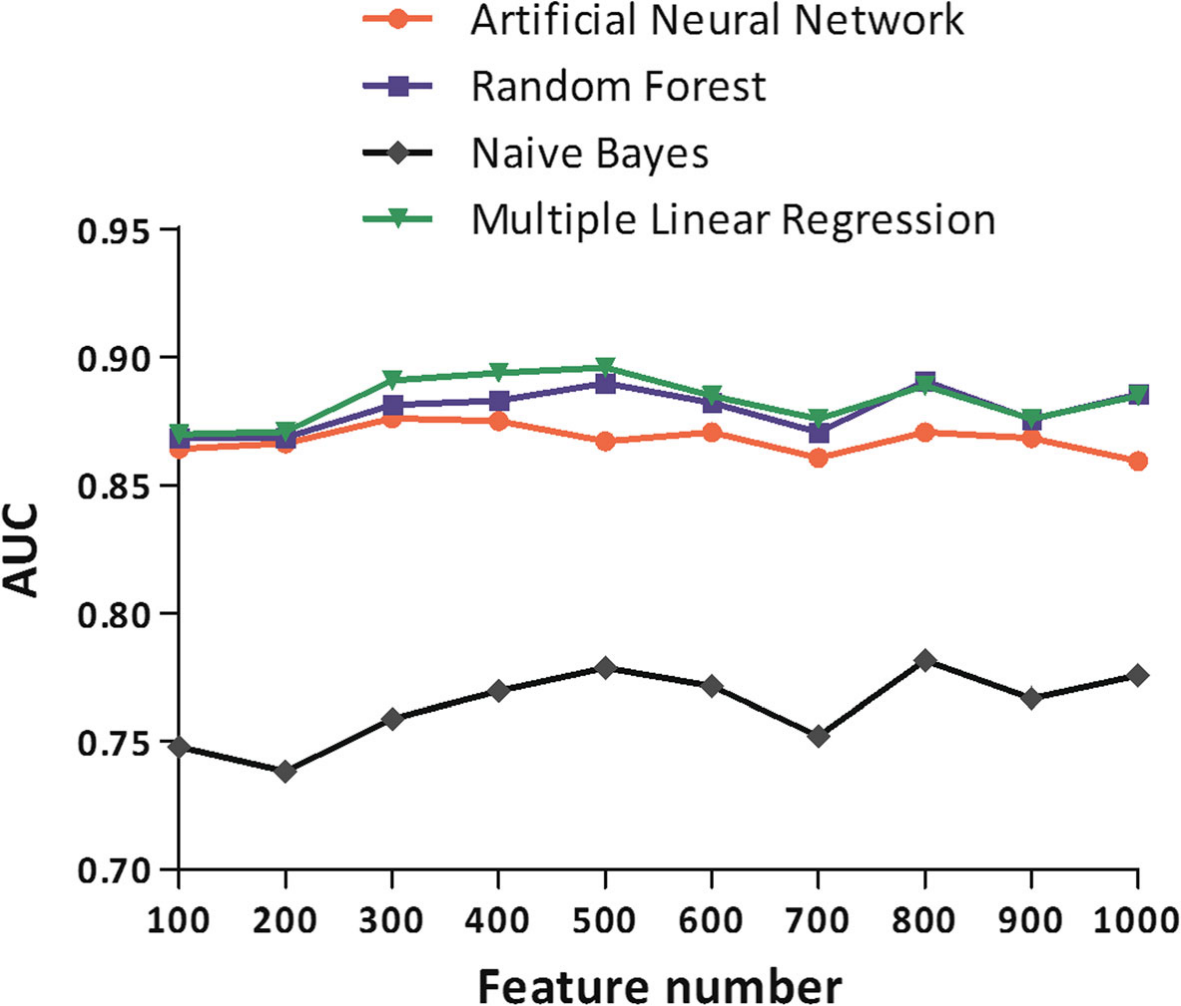
Performances (AUC) of four machine learning algorithms with the increasing number of features. The four machine learning algorithms are artificial neural network, random forest, naïve Bayes and multiple linear regression. Their performances were calculated by 20-fold cross-validation. Of the four algorithms, multiple linear regression model using 500 features showed the best performance (AUC = 0.897)

We constructed prediction models using four different machine learning algorithms (neural network, multiple linear regression, naïve Bayes, and random forest) with increasing number of features from 100 to 1000. Their performances were evaluated by 20-fold cross-validation as described in ***Methods***. Their AUCs with respect to the number of features used are shown in Fig. 2. The four algorithms showed high performances and multiple linear regression showed the highest performance when 500 features were used (AUC = 0.896). For the multiple linear regression, when the number of features was over 500, the AUC decreased slowly because the model started to over-fit to the training dataset. Thus, we constructed a reaction prediction model using multiple linear regression and 500 features.

Our model predicts which of human enzymes can catalyze a query molecule. Firstly, a query molecule is compared with each of the substrates to generate features (descriptor differences) and the model predicts whether the query molecule and the substrate can be catalyzed by the same enzyme. Thus, for a given enzyme the model generates one or more scores depending on the number of its substrates. For the determination of the reactability with the given enzyme, it was necessary to integrate the individual scores. We evaluated four score-integration methods: simple arithmetic mean, simple maximum, probability-based method [21] and our own score-integration method. We compared the performances of the score-integration methods. As explained in ***Methods*** and as shown in Table 2, our score-integration method showed better performance than other methods.

**Table 2:** Performance (AUC) results of four different score-integration methods

Simple average	Simple maximum	Probability-based method^a^	Our method
0.842	0.877	0.884	0.896

^a^Probability-based method is expressed as $\mathrm{S}=1-\prod \limits_i\left(1-{S}_i\right)$, meaning the probability any of the given query-substrate pair is reacted by the same enzyme

**Fig. 3:**
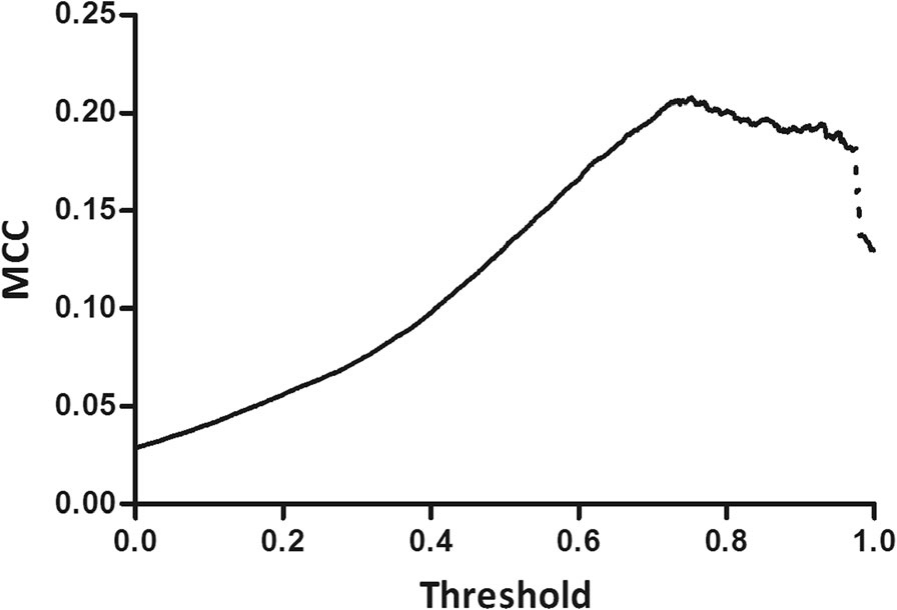
MCC with respect to threshold. To find optimal threshold, Matthews correlation coefficients (MCC) were calculated with an increasing threshold from 0 to 1. MCC was gradually improved with increasing threshold, and began to drop at 0.75. Accordingly, 0.75 was set as a threshold to optimize our model

To further improve the prediction model, the cutoff of integrated score to determine whether a query molecule is catalyzed by a given enzyme was optimized. As the threshold for integrated score increases, the Matthew’s correlation coefficient (MCC) increases. Since most of the data used in the training was biased to negative data (non-reaction), MCC is an appropriate index to show an accuracy of imbalanced dataset. When the threshold was over 0.75, the MCC started to decrease (Fig. 3). Therefore, the threshold of 0.75 was used in our model to determine whether a query molecule is catalyzed by a given enzyme. When this threshold was applied to the training dataset, the model showed a specificity of 0.975, sensitivity of 0.527, and MCC of 0.208 (Table 3).

**Table 3:** Performance results of with a threshold of 0.75

	SEN	SPE	MCC	PPV
Training dataset	0.527	0.975	0.208	0.088
Test dataset	0.171	0.976	0.106	0.089

### Evaluation of the constructed model

We further evaluated our model with a new test dataset that was not used in the training. A test dataset was constructed using DrugBank database and reactions included in the training dataset were removed. The test dataset includes 172 enzymes and 872 substrates. The constructed in silico model was applied to the 872 substrates and predicted which enzymes can catalyze the substrates. The resulting performances are shown in Table 3. Even a new test dataset was used, the model showed reliable performances.

### Performance comparison with other tools

We compared the performance of our model with other tools: admetSAR and deepDTI. The admetSAR predicts the substrate probability of a query molecule for CYP2C9, CYP2D6 and CYP3A4. It should be noted that the admetSAR is a specialized predictor for CYP enzymes, while our model predicts general enzyme-substrate reactability. We used the same test dataset used to evaluate our model. As admetSAR predicts the reactability only with CYP450 enzymes, we also evaluated our model only for CYP450 enzymes. The admetSAR showed a sensitivity of 0.331, specificity of 0.760, and MCC of 0.100. Our model showed a sensitivity of 0.213, specificity of 0.944, and MCC of 0.234. Our method showed significantly higher performance than the admetSAR in predicting molecule-CYP450 reactions.

We also compared our model with a deep-learning-based drug-target interaction prediction tool, deepDTI. As the publicly available deepDTI software requires training step with our own dataset, we trained the tool with the training dataset we used for our model. The performance of deepDTI on the test dataset was significantly low: sensitivity of 0.578, specificity of 0.424, and MCC of 0.0003. The low performance could result from the extreme imbalance in our training and test datasets.

### Further evaluation with literature data

We further evaluated our in silico model with new enzyme-substrate reactions obtained from the literature. There are reports in which non-natural molecules were used for enzyme reactions and thus we used the non-natural molecules (*p*-nitrophenyl acetate, methyl salicylate, *p*-nitrobenzoic acid methylester, tamoxifen and agmatine [10, 25, 26]) for this evaluation. As a result, our model successfully predicted four out of the five chemicals. All reactions predicted by our model are listed in Table 4.

**Table 4:** Top five proteins predicted to interact with the five molecules obtained from the literature^a^

Substrate	UniProt Accession	Enzyme Name
*p*-nitrophenyl acetate	P00915	Carbonic anhydrase 1
	**O00748**	**Cocaine esterase**
	Q14524	Sodium channel protein type 5 subunit alpha
	Q9UI33	Sodium channel protein type 11 subunit alpha
	Q9Y5Y9	Sodium channel protein type 10 subunit alpha
	O60774	Putative dimethylaniline monooxygenase [N-oxide-forming] 6
	Q15166	Serum paraoxonase/lactonase 3
*p*-nitrobenzoic acid methylester	O60774	Putative dimethylaniline monooxygenase [N-oxide-forming] 6
	P00915	Carbonic anhydrase 1
	Q15166	Serum paraoxonase/lactonase 3
	Q14524	Sodium channel protein type 5 subunit alpha
	Q9UI33	Sodium channel protein type 11 subunit alpha
	Q9Y5Y9	Sodium channel protein type 10 subunit alpha
methyl salicylate	Q14524	Sodium channel protein type 5 subunit alpha
	Q9UI33	Sodium channel protein type 11 subunit alpha
	Q9Y5Y9	Sodium channel protein type 10 subunit alpha
	O60774	Putative dimethylaniline monooxygenase [N-oxide-forming] 6
	**O00748**	**Cocaine esterase**
	Q15166	Serum paraoxonase/lactonase 3
	Q01959	Sodium-dependent dopamine transporter
	Q9NUW8	Tyrosyl-DNA phosphodiesterase 1
tamoxifen	O15554	Intermediate conductance calcium-activated potassium channel protein 4
	**P08684**	**Cytochrome P450 3A4**
agmatine	Q96F10	Diamine acetyltransferase 2
	Q9H015	Solute carrier family 22 member 4
	P21673	Diamine acetyltransferase 1
	Q8NE62	Choline dehydrogenase, mitochondrial
	P19623	Spermidine synthase
	**Q9UM01**	**Solute carrier family 7 member 7**

^a^Known interactions are highlighted in bold

## Discussion

We constructed a model to predict which of human enzymes can catalyze the query molecule. As shown in Table 2 and Table 3, the model showed overall high performances even when evaluated with a test dataset: sensitivity of 0.171, specificity of 0.976, MCC of 0.106 and PPV of 0.089. The model showed low PPV on test dataset, and which resulted from the large imbalance of the dataset biased to negative data. When training, 1,764,381 all possible substrate-substrate pair combinations were constructed, and only 11,492 (0.7%) pairs were positive (they are catalyzed by the same enzymes) while the other 1,752,889 (99.3%) were negative (they do not share enzymes). Due to the extreme bias to negative data, it was challenging to predict positive cases and this explains the relatively low sensitivity and PPV. Generally, when negative data size is extremely large, the performance of predicting true positives decreases. On the other hand, when the negative data size is reduced, the performance increases [27].

Our model showed higher performance when compared with previous tools for substrate prediction: admetSAR and deepDTI. It should be noted that the admetSAR is a specialized ADMET prediction tool specific to CYP enzymes, and deepDTI is for the prediction of drug-target interaction. Instead, our method predicts substrate-enzyme reactions, which is not restricted to CYP enzymes and drug targets. Therefore, it may not be fair to compare performances of the specialized tools with our generalized model. Nevertheless, our method showed higher performance than the admetSAR and deepDTI. The deepDTI was evaluated on the test dataset and showed MCC of 0.0003, while our model showed MCC of 0.106. As the admetSAR was developed to predict substrates of CYP enzymes, for fair comparison we used only the substrates of CYP enzymes from the test dataset for evaluation. The MCCs of our model and admetSAR were 0.234 and 0.100, respectively. These results indicate that our model can be used to for practical prediction of substrate-enzyme reactions.

Predictability of our model was further proved using five query compounds found from the literature. Of the five molecules, as shown in Table 4, three molecules (*p*-nitrophenyl acetate, methyl salicylate, and *p*-nitrobenzoic acid methylester) are substrates of cocaine esterase. Their predicted scores for cocaine esterase were 0.847, 0.787, and 0.719, respectively. As we set the threshold as 0.75, *p*-nitrophenyl acetate and methyl salicylate were successfully predicted to react with cocaine esterase enzyme. Our model also predicted that these three molecules could react with serum praxonase/lactonase 3 that mediates the hydrolysis of phenyl acetates. Since methyl salicylate and *p*-nitrophenyl acetate contain phenyl acetate or similar moiety, it is feasible for the two molecules to react with serum praxonase/lactonase 3.

Our model was also used to predict the potential enzymes for tamoxifen that is known to be a substrate of cytochrome P450 3A4. Our model successfully predicted the reaction between tamoxifen and CYP3A4 (score = 0.853). Interestingly, the model also predicted that tamoxifen interacts with a protein with a higher score than CYP3A4, intermediate conductance calcium-activated potassium channel protein 4 (KCNN4, score = 0.872). Although KCNN4 is a potassium transporter, in HMDB KCNN4 was annotated as an enzyme and quinine was assigned as its substrate. However, quinine is an inhibitor of the KCNN4 transporter [28]. Therefore, the annotation for KCNN4 in HMDB was wrong. However, interestingly our model predicted tamoxifen is a potential interacting molecule with KCNN4. We could also find a supporting indirect evidence that tamoxifen affects the function of a calcium-activated potassium channel in mouse [29]. This result demonstrates that our model can predict new chemical compounds that can interact with a query enzyme and interestingly the prediction can be applied to substrates as well as inhibitors/activators.

Solute carrier family 7 member 7 (SLC7A7) is not a metabolic enzyme but a transporter of arginine. However, this transporter was deposited in HMDB and thereby was included in our training dataset. Due to the interaction between SLC7A7 and arginine, our model predicted that agmatine can be a potential chemical compound to be transported by SLC7A7 (score = 0.840), and we could find a supporting literature evidence for the interaction [26]. Interestingly, our model also predicted that SLC22A4, a member of solute carrier family, is able to transport agmatine as well (score = 0.948). Although there is no evidence about their interaction, agmatine is known to be transported by other members of solute carrier family 22, SLC22A1 and SLC22A3 [30], and therefore the SLC22A4 would transport agmatine.

Our model successfully predicted the interaction of SLC7A7 and agmatine, and SLC22A4 and agmatine. This proves that our model can predict general interactions between molecules and proteins, and not limited to substrates and enzymes.

## Conclusion

In this study, we developed an in silico model to predict which of human enzymes can catalyze a query molecule. The model was based on the assumption that if the physico-chemical properties expressed as descriptors of a query compound and a known substrate were similar, they would be catalyzed by the same enzyme. Our model is not limited to substrate-enzyme interactions, but can be generalized to the interactions between molecules and transporters, and interactions between inhibitors and drug targets.

There are an increasing number of reports that drugs can be modified by enterobacteria in human gut [31]. The same principle underlying in our model could also be applied to predict the enzymatic reactions mediated by human gut bacteria. In addition, the prediction can be used with various other information, such as the distribution of enzymes in human tissues. With this information, it would be possible to predict tissue-specific enzymatic reactions and to analyze the effect of biotransformation. Furthermore, it could be possible to predict unknown routes of metabolic pathways by predicting undiscovered reactions. Consequently, our in silico model should be a useful tool to screen drug candidates to computationally assess drug modifications and to predict unknown chemical reactions in biochemical studies.

## Abbreviations

ADMET: Absorption, Distribution, Metabolism, Excretion and Toxicity
AUC: Area Under ROC Curve
BRENDA: BRaunschweig ENzyme DAtabase
HMDB: Human Metabolome Database
InChI: IUPAC International Chemical Identifier
MCC: Matthews Correlation Coefficient
PPV: Positive Predictive Value
ROC: Receiver Operating Characteristic
SEN: Sensitivity
SMILES: Simplified Molecular-Input Line-Entry System
SPE: Specificity
